# Serum C-Reactive Protein and Procalcitonin Kinetics in Patients Undergoing Elective Total Hip Arthroplasty

**DOI:** 10.1155/2014/565080

**Published:** 2014-05-04

**Authors:** Sandra Battistelli, Mattia Fortina, Serafino Carta, Roberto Guerranti, Francesco Nobile, Paolo Ferrata

**Affiliations:** ^1^Department of Surgery, University Hospital of Siena, Viale Bracci No. 16, 53100 Siena, Italy; ^2^Orthopaedics and Traumatology Clinic, University Hospital of Siena, Viale Bracci No. 16, 53100 Siena, Italy; ^3^Department of Internal Medicine, University Hospital of Siena, Viale Bracci No. 16, 53100 Siena, Italy

## Abstract

*Background*. The sensitivity and the specificity of different methods to detect periprosthetic infection have been questioned. The current study aimed to investigate the kinetics of C-reactive protein (CRP) and procalcitonin (PCT) in patients undergoing uncomplicated elective total hip arthroplasty (THA), to provide a better interpretation of their levels in noninfectious inflammatory reaction. *Methods*. A total of 51 patients were included. Serum CRP and PCT concentrations were obtained before surgery, on the 1st, 3rd, and 7th postoperative days and after discharge on the 14th and 30th days and at 2 years. *Results*. Both markers were confirmed to increase after surgery. The serum CRP showed a marked increase on the 3rd postoperative day while the peak of serum PCT was earlier, even if much lower, on the first day. Then, they declined slowly approaching the baseline values by the second postoperative week. PCT mean values never exceed concentrations typically related to bacterial infections. *Conclusions*. CRP is very sensitive to inflammation. It could be the routine screening test in the follow-up of THA orthopaedic patients, but it should be complemented by PCT when there is the clinical suspicion of periprosthetic infection.

## 1. Introduction

In modern total hip arthroplasty (THA), postoperative septic complications are rare. Advancements in implant design, device manufacturing, perioperative protocols, and surgical techniques have led to improved functional outcomes and longevity of THA. Nevertheless, prosthetic joint infection is one of the most serious complications, occurring in 0.3 to 1.7% of hip arthroplasty [1–3]. Its correct diagnosis is crucial for adequate surgical treatment. In the early postoperative days, its detection is particularly difficult and currently it can be established on the basis of several concurring parameters such as clinical presentation, laboratory markers, imaging study, and microbiologic testing. C-reactive protein (CRP) and, more recently, procalcitonin (PCT) are two laboratory tests of considerable usefulness in clinical practice. CRP is the most available and widely used. CRP is an acute phase protein produced predominantly by hepatocytes in response to interleukin-6 trigger. Its serum concentration is very low in healthy subjects but it increases rapidly in cases of inflammation, infection, and traumatic injury. It has a long half-life and stable levels with negligible circadian variation [4–6]. CRP is used to monitor the postoperative course in surgical trauma following orthopaedic implants and to detect prosthetic infection.

PCT is a marker of infection. It has been found to circulate at very low concentrations in normal serum, presumably produced by the same cells where calcitonin is synthetized [7–9]. Clinical studies in human have shown that the highest serum PCT values occur in patients with sepsis [10]. They are also increased in inflammatory conditions, such as extensive surgery [11–13]. Postoperatively some unspecific or trauma related induction of PCT has been reported, where detailed analysis is still missing. It follows that early detection of postoperative bacterial complications is extremely difficult.

In the present study, we analyzed the spontaneous postarthroplasty kinetics of PCT and CRP in a cohort of consecutive patients admitted to our institution for primary hip replacement. The aim of the study was to investigate the “normal” or “physiological” pattern of the two biochemical tests and their correlations over the first month after uncomplicated elective THA, as a basis to reach an appropriate interpretation of their levels in postsurgical noninfectious inflammation.

## 2. Materials and Methods

We prospectively included a total of 51 patients (25 males, mean age 70 years, range 42–83; 26 females, mean age 70 years, range 56–86) with primary hip osteoarthritis, admitted to the Orthopaedic and Traumatology Clinic of the University Hospital of Siena, from June 2010 to December 2011 for total hip replacement due to osteoarthritis. The exclusion criteria were as follows: other underlying inflammatory or infectious disease, cardiac, renal (creatinine clearance < 50 mL/min), and respiratory failure, malignancy, and operative procedure within 3 months before admission. There were no postoperative complications during the inpatient stay, in the first month after operation, and at the final follow-up of two years. In all cases, the absence of infection was confirmed by clinical symptoms and signs, laboratory findings, and radiographic studies including the anteroposterior projection of the pelvis and the axial projection of the operated hip. Any radiolucent line around the prosthesis was assessed according to the three DeLee and Charnley zones [14] for the cup and to the Gruen zones for the stem [15]. We defined components as radiologically loose if they had migrated 2 mm or more. Radiolucent lines of 2 mm or more in any zone adjacent to the coated portion of the implant and any lytic lesion or subperiosteal formation of new bone around the distal stem were also considered to indicate loosening. The final radiographs were graded according to the criteria of Engh et al. for the type of fixation [16].

To evaluate the clinical presence of the periprosthetic joint infection, we followed the criteria proposed by the Musculoskeletal Infection Society (MSIS). Based on the proposed criteria, a definite diagnosis of prosthetic joint infection can be made when the following conditions are met:

1. a sinus tract is communicating with the prosthesis;
2. a pathogen is isolated by culture from two separate tissue or fluid samples obtained from the affected prosthetic joint; or
3. four of the following six criteria exist:
  - elevated serum erythrocyte sedimentation rate (ESR) or serum C-reactive protein (CRP) concentration,
  - elevated synovial white blood cell (WBC) count,
  - elevated synovial neutrophil percentage (PMN%),
  - presence of purulence in the affected joint,
  - isolation of a microorganism in one culture of periprosthetic tissue or fluid,
  - greater than five neutrophils per high-power field in five high-power fields observed from histologic analysis of periprosthetic tissue at 400 times the magnification.

THA were performed by the same surgeon using the ALDI (anterior lateral decubitus intermuscular) approach. This is our tissue sparing interpretation of the Smith-Petersen approach. We have developed this approach to unify the advantages of the anterior approach and those of the patient's lateral position. The result is a complete intermuscular and internervous approach that allows us to preserve all the hip muscles and tendons without changing the surgeon's point of view during the acetabular preparation. This approach has become our standard in THA due to excellent results since 2009. The operative time was about 51′ (range 45–60). General anaesthesia was performed in all cases. Prophylactic antibiotics (cefazolin 1 g) were given 1 h before surgery and every 8 h for 36 h postoperatively. All patients received antithrombotic prophylaxis with fondaparinux, 2.5 mg per day for at least 35 days, starting from 6–8 hours to 12 hours after surgery. Fluid therapy and physiotherapy were delivered according to the usual local standard of care. The mean haemoglobin loss on the 5th day was 3.4 g/dL. Blood transfusions (600 cc of packed red blood cells) were performed in 10 patients (17.8%), due to haemoglobin values <8.0 g/dL (9.0 g/dL in case of heart disease). The mean hospital stay was 7 days (range 6–10). Blood samples for determination of CRP and PCT were obtained at baseline (the day before the operation), on the 1st, 3rd, and 7th postoperative days, after discharge on the 14th and 30th days, and at the final follow-up at two years. The study was approved by the local Ethics Committee and informed written consent was obtained from all participants. Peripheral blood samples for laboratory investigations were collected from a cubital vein to VacutainerTM vacuum test tubes after overnight fasting. CRP levels were analyzed by high-sensitivity immunoturbidimetric technique on the Roche/Hitachi Modular System, using 3rd-generation CRP Tina-Quant reagent. The functional sensitivity was 0.042 mg/dL and the normal values were set to less than 0.5 mg/dL. PCT levels were quantitatively measured using an Electrochemiluminescent Immunoassay (E.C.L.I.A., BRAHMS AG, Hennigsdorf, Germany) on the Cobas e601 analyzer (Roche, Hitachi). The functional sensitivity was 0.02 ng/mL and the normal values were set to less than 0.046 ng/mL (95th percentile). Since normality of data distribution could not be statistically verified by the Kolmogorov-Smirnov test, the nonparametric Kruskal-Wallis with Dunn's Multiple Comparison posttest was performed using GraphPad Prism version 4.00 for Windows (GraphPad Software, San Diego, California, USA, http://www.graphpad.com/). To assess correlation between the temporal pattern of CRP and PCT the Spearman correlation was used. A statistically significant difference was inferred when *P* was <0.05.

## 3. Results

**Figure 1:**
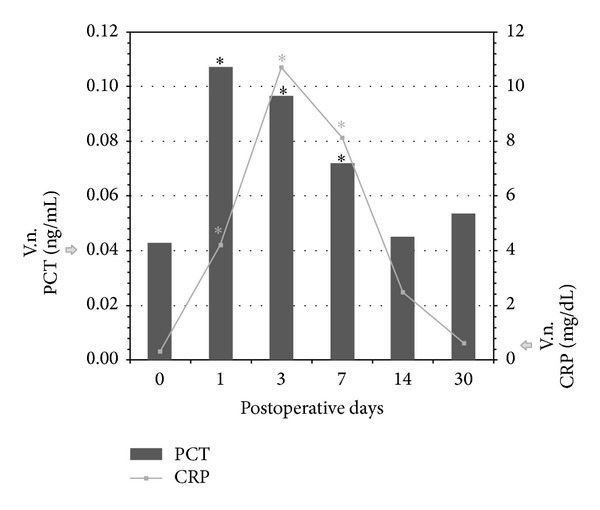
Mean postoperative PCT and CRP levels in patients after hip surgery. The horizontal axis represents postoperative days and the vertical axis represents the respective concentration of PCT and CRP. *P value significantly different from baseline.

The preoperative CRP mean serum levels were roughly in the normal range (0.32 ± 0.24 mg/dL) in our group of patients. Thereafter, the reference curve describing the induction of CRP during the first postoperative month revealed a rapid increase in levels of CRP (13-fold higher than baseline mean values) on the 1st day, with further increase, up to a peak of 33 times the mean normal levels (10.7 ± 4.3 mg/dL) on the 3rd day (Tables 1 and 2). Then, it began to drop. It was still significantly elevated (26-fold higher than the mean baseline) but statistically different from the peak day, on the 7th day. From the 14th day, its mean values were not statistically different from the baseline. On the 30th postsurgical day, mean concentrations of CRP returned close to the height of normal range. In contrast, the temporal course of PCT induction was earlier, although much lower, than that of CRP (Tables 1 and 3). Levels of PCT increased rapidly, compared to the baseline concentrations (0.04 ± 0.02 ng/mL), reaching a peak on the first postsurgical day (0.12 ± 0.12 ng/mL, 3-fold higher than the mean preoperative levels) but not exceeding concentrations typically related to bacterial infections [11, 12]. On the 3rd and 7th days, its levels, despite being in progressive decline, were not statistically different from the peak day. Then, the PCT declined significantly, like the CRP. On the 14th day, the concentrations of PCT normalized while on the 30th day they were close to the height of normal range (0.05 ± 0.01 ng/mL) (Figure 1). A very high interindividual variation was observed in the first postsurgical week in both values of CRP and PCT indicating a great range and overlap of values. The large standard deviations decreased strongly by the 14th day. Preoperative and postoperative values of both markers, CRP and PCT, correlated inconsistently (Tables 2 and 3). No significant correlation was found between the kinetics of the two biochemical markers (Spearman's *r* = 0.84).

No patients met the criteria proposed by the Musculoskeletal Infection Society (MSIS) at any follow-up control.

## 4. Discussion

Serum C-reactive protein and more recently PCT have been proposed to monitor short- and long-term infection in the setting THA, but appropriate interpretation of their levels in the early postoperative period is not easy, because of the associated inflammatory condition. Previous studies have reported that, in uncomplicated THA, the CRP levels increase rapidly after surgery, up to a peak within 2-3 days. Thereafter, the levels fall and slowly become negative, in a range encompassing 3-4 to 6–8 weeks [17–20]. According to the literature, in our group of patients, the CRP mean serum concentrations peaked on the 3rd day after surgery, when a sharp increase was recorded (mean value 10.7 mg/dL). On the 7th postoperative day, they were statistically lower than the peak day but still significantly above the baseline values. Then, by the end of the second postoperative week, the PCR dropped significantly and 4 weeks after the surgical procedure its mean values were close, but not equal, to the height of the preoperative values, indicating a persistent certain degree of inflammatory reaction. As documented by the high interindividual variation (from 2.5 to 24 mg/dL), for each patient, the peak concentration of CRP could vary, but the level then fell with a similar pattern and, based on our findings, any upward trend in the CRP levels might point to the presence of a complication. However, it should be emphasized that serum CRP is a nonspecific marker of inflammation and infection. It is not very helpful in the diagnosis of deep infection of an implant since its sensitivity is not 100% and some patients with an infected implant might present with normal CRP values. Low grade infections or encapsulated infections may result in less intensive systemic reaction and are sometimes associated with normal laboratory markers [21, 22]. Moreover, to avoid false positive results, estimation of the level of PCT is suggested in patients with elevated CRP. Serum PCT has been introduced as a more accurate marker for general bacterial infection, but trauma or surgery may also result in its transient elevation. Literature concerning postoperative PCT is not poor but it is not specific enough for evaluation of specific surgical intervention; moreover, it focuses only on a limited range of time [13, 23]. Results from these studies vary greatly, due to the different surgical procedures and protocol, diverse characteristics of the patients, and insufficient sensitivity of the PCT assay [24–28]. Many authors deny the utility of serum PCT sampling for the diagnosis of localized osteoarticular infection. In a retrospective study from infected adult orthopaedic patients, the postoperative serum PCT reached the peak levels on the first postsurgical day only in half of the patients. Then, it fell quickly within normal range on the second day, despite ongoing infection, during the following days. The authors concluded that PCT does not seem to be better than the less expensive CRP in the follow-up of infected patients [29]. A further prospective study evaluated the role of laboratory markers in the diagnosis of deep implant infection in patients with a total knee revision or total hip arthroplasty. CRP had high sensitivity while PCT was very specific but less sensitive. The authors suggested that the combination of the two markers could be useful in identifying patients with true positive CRP [26]. According to previous reports, in the present study, serum PCT reached the peak levels quickly, on the first postsurgical day (0.11 ng/mL) but, thereafter, it declined slowly, following the natural evolution of the postsurgical inflammatory process. As for the CRP, levels of PCT approached baseline only by the second postsurgical week. Notably, the recorded peak levels of PCT were much lower than the levels commonly seen in infectious conditions [10]. As some authors stated [30, 31] in E.C.L.I.A, PCT values >2 ng/mL are strongly indicative of sepsis or severe bacterial infection, while levels below approximately 0.5 ng/mL make these conditions unlikely. Moreover, as well as CRP, the high interindividual variations of serum PCT observed in the first postsurgical day, from moderately high (maximum 0.78 ng/mL) to normal (minimum 0.01 ng/mL), suggest that single or occasional values can be misleading. Early postsurgery determination may not have prognostic value. The moderate elevation of PCT often seen in these occurrences may be independent of any infection. They may become more meaningful later in the course if a clear trend is demonstrated (i.e., decreasing or increasing or unchanging). Serial PCT measurements should then be performed, when indicated, to define its diagnostic and prognostic usefulness.

Serum CRP and PCT are two unspecific serologic markers of inflammation and infection that, in our group of uncomplicated THA patients, increased differently, but with similar trend, in the postoperative days. The increase of the CRP was markedly elevated while that of PCT was earlier but much lower, below the levels usually reported for bacterial infections. They attained the normal values approximately from the second postsurgical week. It follows that any upward trend in their level should suggest a complication: high initial levels, their persistence, or a secondary increase at a later time. In particular, high levels of CRP in the first postoperative week are the normal postoperative course and could be interpreted as infection CRP only if their increase to levels of pathology persists during time. Therefore, the high sensitivity of CRP can be used in the later period after surgery as possible sign of infection (e.g., 2-3 weeks after surgery to 1-2 years or later), although the baseline values should be known.

PCT, very expensive and more specific for infection, should complement the common serologic tests only when there is the clinical suspicion of infection. PCT is useful also in patient with postoperative unknown fever that could be managed with early antibiotic therapy only if its values are high. The high negative predictive values of PCT do not exclude bacterial infection since to date its usefulness in detecting local infection is questionable. Conversely, any increase of PCT above the reference range that we found for the first postoperative days and thereafter any increase may suggest infection. In patients with increase of PCT, diagnosis of infection should be checked and otherwise verified.

## Figures and Tables

**Table 1:** Increased fold of mean of CRP and PCT in comparison to basal levels.

Days	CRP	PCT
0	0.0	0.0
1	13.2	2.7
3	33.7	2.3
7	25.6	1.7
14	7.7	1.1
30	1.9	1.2

**Table 2:** CRP descriptive statistic (all values are expressed in mg/dL).

Days	0	1	3	7	14	30
Minimum	0.06	0.4	2.5	1.95	0.22	0.08
25% percentile	0.14	2.005	8.29	4.455	1.315	0.13
Median	**0.24**	**3.63**	**10.34**	**7.47**	**2.015**	**0.6**
75% percentile	0.435	5.34	13.21	11.45	3.585	0.89
Maximum	1.32	13.82	24.13	19.26	5.63	1.64
Significativity of median variation in comparison to basal level		*P* < 0.001	*P* < 0.001	*P* < 0.001	*P* > 0.05	*P* > 0.05
Mean	**0.3178**	**4.207**	**10.7**	**8.121**	**2.46**	**0.6133**
Std. deviation	0.2369	2.988	4.344	4.377	1.771	0.5004
Std. error	0.03223	0.4104	0.5858	0.6069	0.6263	0.1444
Significativity of mean variation in comparison to basal level		*P* < 0.001	*P* < 0.001	*P* < 0.001	*P* > 0.05	*P* > 0.05
Lower 95% CI of mean	0.2531	3.384	9.522	6.902	0.979	0.2954
Upper 95% CI of mean	0.3824	5.031	11.87	9.339	3.941	0.9312

**Table 3:** PCT descriptive statistic (all values are expressed in ng/mL).

Days	0	1	3	7	14	30
Minimum	0.02	0.01	0.02	0	0.03	0.04
25% percentile	0.02	0.06	0.06	0.05	0.04	0.045
Median	**0.04**	**0.09**	**0.08**	**0.07**	**0.04**	**0.05**
75% percentile	0.05	0.14	0.12	0.08	0.05	0.06
Maximum	0.15	0.78	0.49	0.22	0.07	0.08
Significativity of median variation in comparison to basal level		*P* < 0.001	*P* < 0.001	*P* < 0.001	*P* > 0.05	*P* > 0.05
Mean	**0.04275**	**0.1159**	**0.1002**	**0.07196**	**0.045**	**0.05333**
Std. deviation	0.02515	0.1167	0.07212	0.03995	0.01195	0.01303
Std. error	0.003521	0.01634	0.009907	0.005594	0.004226	0.003761
Significativity of mean variation in comparison to basal level		*P* < 0.001	*P* < 0.001	*P* < 0.001	*P* > 0.05	*P* > 0.05
Lower 95% CI of mean	0.03567	0.08307	0.08031	0.06072	0.03501	0.04506
Upper 95% CI of mean	0.04982	0.1487	0.1201	0.0832	0.05499	0.06161
